# Potential association of rheumatic diseases with bone mineral density and fractures: a bi-directional mendelian randomization study

**DOI:** 10.1186/s12891-024-07496-w

**Published:** 2024-07-05

**Authors:** Chen-xuan Hong, Yan-zheng Pan, Feng-bo Dai

**Affiliations:** 1 Department of Orthopaedics, The Affiliated Cangnan Hospital of Wenzhou Medical University, Wenzhou, Zhejiang 325899 China; 2 Department of Orthopaedics, The Second Affiliated Hospital, Yuying Children’s Hospital of Wenzhou Medical University, Wenzhou, Zhejiang 325027 China

**Keywords:** Mendelian randomization, Rheumatic diseases, Bone mineral density, Fracture

## Abstract

**Background:**

Previous studies have implicated rheumatoid arthritis as an independent risk factor for bone density loss. However, whether there is a causal relationship between rheumatic diseases and bone mineral density (BMD) and fractures is still controversial. We employed a bidirectional Mendelian analysis to explore the causal relationship between rheumatic diseases and BMD or fractures.

**Methods:**

The rheumatic diseases instrumental variables (IVs) were obtained from a large Genome-wide association study (GWAS) meta-analysis dataset of European descent. Analyses were performed for the three rheumatic diseases: ankylosing spondylitis (AS) (*n* = 22,647 cases, 99,962 single nucleotide polymorphisms [SNPs]), rheumatoid arthritis (RA) (*n* = 58,284 cases, 13,108,512 SNPs), and systemic lupus erythematosus (SLE) (*n* = 14,267 cases, 7,071,163 SNPs). Two-sample Mendelian randomization (MR) analyses were carried out by using R language TwoSampleMR version 0.5.7. The inverse-variance weighted (IVW), MR-Egger, and weighted median methods were used to analyze the causal relationship between rheumatic diseases and BMD or fracture.

**Results:**

The MR results revealed that there was absence of evidence for causal effect of AS on BMD or fracture. However, there is a positive causal relationship of RA with fracture of femur (95% CI = 1.0001 to 1.077, *p* = 0.046), and RA and fracture of forearm (95% CI = 1.015 to 1.064, *p* = 0.001). SLE had positive causal links for fracture of forearm (95% CI = 1.004 to 1.051, *p* = 0.020). Additionally, increasing in heel bone mineral density (Heel-BMD) and total bone mineral density (Total-BMD) can lead to a reduced risk of AS without heterogeneity or pleiotropic effects. The results were stable and reliable. There was absence of evidence for causal effect of fracture on RA (95% CI = 0.929 to 1.106, *p* = 0.759), and fracture on SLE (95% CI = 0.793 to 1.589, *p* = 0.516).

**Conclusions:**

RA and SLE are risk factors for fractures. On the other hand, BMD increasing can reduce risk of AS. Our results indicate that rheumatic diseases may lead to an increased risk of fractures, while increased BMD may lead to a reduced risk of rheumatic diseases. These findings provide insight into the risk of BMD and AS, identifying a potential predictor of AS risk as a reduction in BMD.

**Supplementary Information:**

The online version contains supplementary material available at 10.1186/s12891-024-07496-w.

## Introduction

Rheumatic diseases cover a variety of conditions that primarily affect the joints, bones, muscles, and connective tissues in the body [1, 2]. There are more than 100 different types of rheumatic diseases, such as rheumatoid arthritis (RA), systemic lupus erythematosus (SLE), ankylosing spondylitis (AS), osteoarthritis, psoriatic arthropathy and inflammatory spondylitis and so on. They represent a variety of diseases with different characteristics. Various risk factors, including some controllable factors such as obesity and osteoporosis, are associated with the development of these rheumatic diseases [3–5]. Fracture is a common complication of rheumatic diseases. Rheumatic diseases have been reported to cause low bone mass with periarticular osteoporosis, malnutrition, and osteomalacia due to malabsorption [6]. Inflammatory rheumatic diseases, in particular, may lead to an increased risk of fractures [7, 8]. Rheumatic diseases in children, such as juvenile idiopathic arthritis, can affect the skeletal system, leading to reduced bone mineral density (BMD) and a high risk of fragility fractures in childhood [9].

Ankylosing spondylitis (AS) is a chronic autoimmune disease marked by persistent inflammation and the development of new bone in the central axial skeleton and sacroiliac joints [10, 11]. It also extends to peripheral joints and may involve extra-articular areas. These developments can lead to inflammatory low back pain and limitations in spinal mobility. Additionally, individuals with AS experience a decrease in BMD over the course of the disease. Research has consistently shown that BMD reduction is an early occurrence in the progression of ankylosing spondylitis [12]. Rheumatoid arthritis (RA) is an autoimmune disorder primarily targeting the joints. It manifests as chronic inflammation, resulting in joint pain, swelling, stiffness, and the erosion of joint tissues [13, 14]. This inflammatory cascade can also adversely affect bone health, including BMD. Numerous studies have demonstrated that RA is linked independently to a heightened risk of osteoporosis, leading to a significant increase in fracture risk and BMD decline [15, 16]. Systemic lupus erythematosus (SLE) is an autoimmune disease capable of impacting multiple organs and bodily systems, including the musculoskeletal system [17]. The connection between SLE and BMD is significant, as SLE can contribute to decreased BMD levels, elevating the risk of osteoporosis and fractures [18, 19]. SLE patients exhibited significantly lower BMD measurements at the lumbar spine and hip when compared to healthy controls matched for sex, age, and geographical location, and similar measurements to those of RA patients [18].

BMD is a metric employed to evaluate the mineral content in specific bone regions, commonly the spine, hip, or forearm. It provides an indication of bone density and strength, which are crucial for skeletal health [20]. Many rheumatic diseases can lead to a decline in bone density, heightening the susceptibility to osteoporosis and fractures [21, 22]. However, observational studies have limitations. They may not account for unmeasured or unknown confounding variables and typically cannot definitively establish a direct cause-and-effect relationship between exposure and outcome unless the study design meticulously ensures temporal and causal sequencing. In the context of most observational studies on BMD and rheumatic diseases, uncertainty persists regarding whether diminished BMD escalates the risk of developing the condition or if individuals with a rheumatic ailment are more predisposed to fractures. Additionally, it’s plausible that the connection between BMD and rheumatic conditions arises from an underlying lifestyle or environmental factor that elevates the risk for both conditions. So far, there is no conclusive evidence whether there is a causal relationship between rheumatic diseases and BMD or whether an increase in BMD can reduce the risk of rheumatic diseases.

Mendelian randomization (MR) is an approach used to probe causal relationships, effectively sidestepping the aforementioned limitations by employing genetic variants as exposure instruments [23, 24]. Recently, an MR study was used to explore the causality between AS and osteoporosis [12]. In the present study, we utilized data from publicly available genome-wide association studies (GWAS) to investigate the causal relationship between rheumatic diseases (AS, RA, and SLE) and BMD or fracture. The initial phase involved examining whether AS, RA, and SLE exert causal effects on BMD measurements or fracture. Subsequently, in the second phase, we assessed whether BMD measurements or fracture are causally linked with AS, RA, and SLE.

## Materials and methods

### Study design

**Fig. 1:**
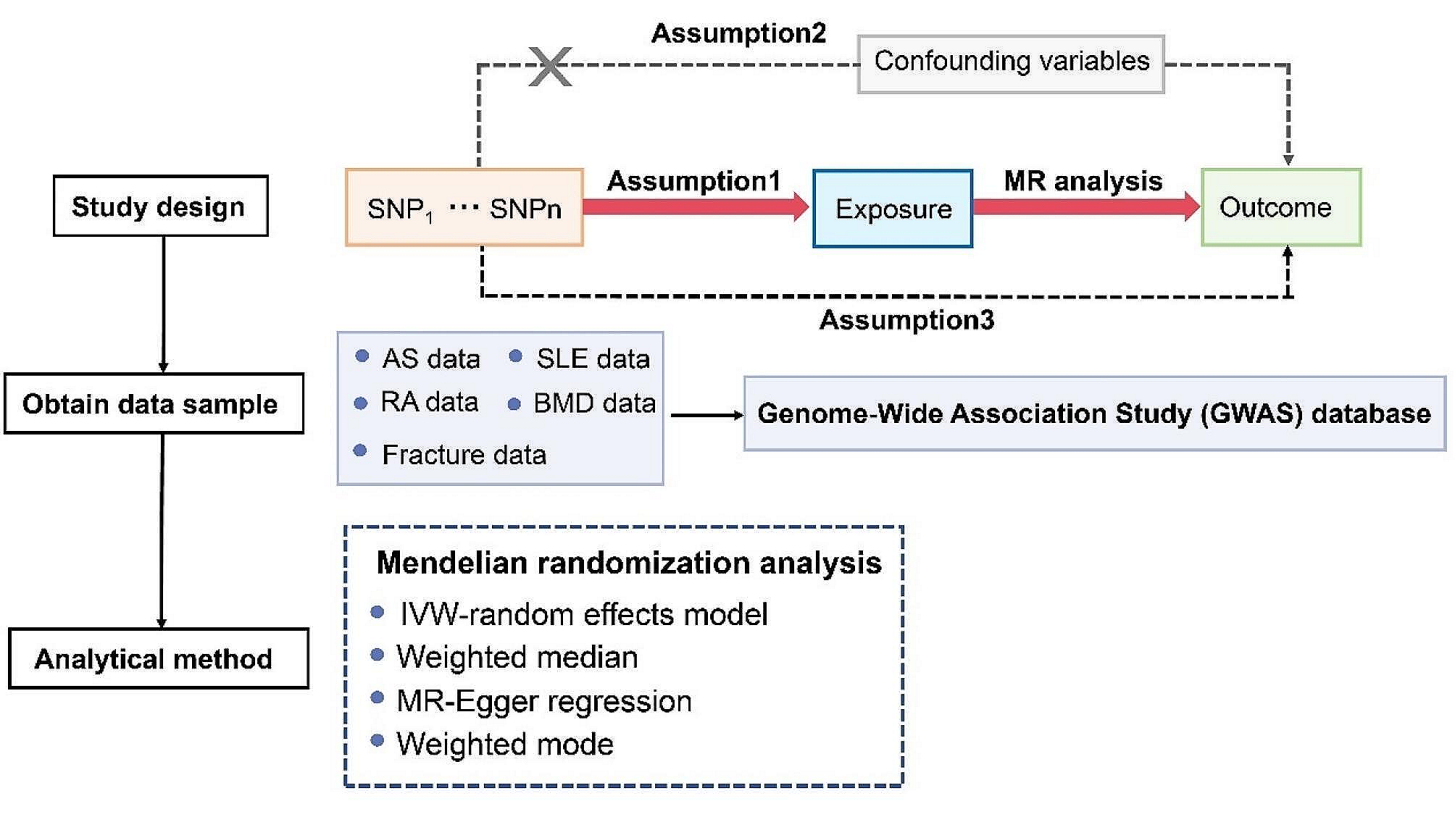
The schematic diagram of Mendelian randomization (MR). Three assumptions should be met, as follows: Assumption 1: The SNPs should be closely related to exposures; Assumption 2: The IVs selected are supposed to be independent of confounders; Assumption 3: SNPs should　influence the outcomes just through the exposure. (IVs, instrumental variables; SNPs, single-nucleotide polymorphisms)

The assumptions underlying the MR analysis of the causal relationship between rheumatic diseases and BMD or fracture include: Assumption 1: The selected single nucleotide polymorphisms (SNPs) should exhibit a robust correlation with the exposure; Assumption 2: The selected SNPs should not be associated with the outcome through confounding variables; Assumption 3: The selected SNPs are anticipated to influence outcomes through exposure, rather than through a direct association (Fig. 1). All data utilized in this study were sourced from publicly available databases or pre-existing publications, obviating the need for additional ethical approval.

### Data sources

Data for the two-way MR analysis were obtained from the Genome-Wide Association Study (GWAS) database (https://gwas.mrcieu.ac.uk/). Instrumental variables for rheumatic diseases were as follows: AS included 22,647 samples (number of cases: 9,069, number of controls: 1,550) from Europe with a total of 99,962 SNPs; RA included 58,284 samples (number of cases: 14,361, number of controls: 43,923) from Europe with a total of 13,108,512 SNPs; SLE included 14,267 samples (number of cases: 5,201, number of controls: 9,066) from Europe with a total of 7,071,163 SNPs. BMD data encompassed measurements for the total bone density and heel bone density sourced from the GWAS database [12]. Specifically, the sample sizes for each category were as follows: whole body: 56,284 samples with a total of 16,162,733 SNPs; heel: 265,627 samples with a total of 9,851,867 SNPs. Fracture data included occurrences in the femur, pelvis, lumbar spine, and forearm, obtained from the GWAS database [25]. Sample sizes for each category were as follows: forearm: 215,724 samples (number of cases: 9,956, number of controls: 205,768) with a total of 16,380,460 SNPs; femur: 215,443 samples (number of cases: 3,983, number of controls: 211,460) with a total of 16,380,458 SNPs; lumbar spine and pelvis: 215,698 samples (number of cases: 2,859, number of controls: 212,839) with a total of 16,380,457 SNPs (Supplementary Table 1).

### Analytical method

Mendelian randomization analysis was conducted using the R programming language, specifically with the TwoSampleMR package version 0.5.7. In this two-sample MR analysis, genome-wide significant SNPs (with *P* < 5 × 10^− 8^) were employed as instrumental variables to investigate the causal impacts of AS, RA, and SLE on BMD and fracture risk. To mitigate the influence of linkage disequilibrium, a clumping process (R^2^ < 0.001, window size = 10,000 kb) [26] was applied to the European samples from the 1000 Genomes Project. This study utilized multiple methods including inverse variance weighted (IVW), weighted median, MR-Egger, and weighted mode approaches to delve into the causal relationships between AS, RA, and SLE, and BMD or fracture risk. The MR Egger regression intercept was employed to assess pleiotropy.

### Statistical analyses

This study adopted the IVW, weighted median, MR-Egger, and weighted mode methods to explore the causal relationships between AS, RA, and SLE and BMD or fractures. The MR Egger regression intercept was used to evaluate pleiotropy, and when *p* > 0.05, it was considered that there was no pleiotropy. Heterogeneity was assessed using Cochrane’s Q statistic, and Q_pval > 0.05 was considered to have no heterogeneity. Positive results ensured that IVW was statistically significant (*p* < 0.05), and that the β directions of IVW, MR Egger, and Weighted median were consistent. Positive results at least ensure that IVW is significant (*p* < 0.05), and the β directions of IVW, MR Egger, and Weighted median are consistent.

In addressing the multiplicity of hypothesis tests conducted within our study, we implemented rigorous statistical correction methods to control the risk of Type I errors. Specifically, we applied both the Bonferroni correction and the Benjamini-Hochberg false discovery rate (FDR) controlling procedure. The Bonferroni correction was utilized to adjust the significance thresholds for each individual test, setting the corrected alpha at alpha = 0.05/number of tests, which stringently controls the family-wise error rate. Concurrently, the Benjamini-Hochberg procedure was employed to control the FDR, offering a balance between discovery and error rate, which is particularly advantageous in studies with a high number of simultaneous hypothesis tests.

## Results

### Casual relationship between AS and BMD or fractures

To assess the potential causal impact of rheumatic diseases on BMD and fracture risk, we first examined the casual relationship between AS and BMD or fractures by a two-sample MR analysis. In our study, we included 23–25 independent single nucleotide polymorphisms (SNPs) as instrumental variables (IVs) for AS. The results from weighted median, IVW, and MR-Egger regression tests collectively indicate that there is no significant association between AS and either BMD or fractures (Table 1).

**Table 1:** The Mendelian randomization (MR) analysis results of AS on fracture of forearm, fracture of femur, fracture of lumbar spine and pelvis, Heel-BMD and Total-BMD.

Outcomes		Fracture of forearm (nSNPs = 24)	Fracture of femur (nSNPs = 24)	Fracture of lumbar spine and pelvis (nSNPs = 24)	Heel-BMD (nSNPs = 25)	Total-BMD (nSNPs = 25)
IVW	OR(95%CI)	1.042 ( 0.885–1.227 )	1.114 ( 0.905–1.371 )	1.192 ( 0.927–1.532 )	0.994 ( 0.948–1.043 )	0.984 ( 0.933–1.037 )
	*p*	0.621	0.309	0.171	0.809	0.539
MR Egger	OR(95%CI)	1.196 ( 0.904–1.583 )	1.364 ( 0.952–1.954 )	1.783 ( 1.176–2.704 )	1.025 (0.902–1.165 )	0.922 ( 0.843–1.009 )
	*p*	0.224	0.105	0.012	0.706	0.090
Weighted median	OR(95%CI)	1.063 ( 0.874–1.292 )	1.198 ( 0.901–1.594 )	1.308 ( 0.938–1.825 )	1.013 ( 0.962–1.067 )	0.972 ( 0.903–1.047 )
	*p*	0.543	0.215	0.114	0.619	0.456
Weighted mode	OR(95%CI)	1.07 ( 0.886–1.292 )	1.196 ( 0.898–1.594 )	1.351 ( 0.966–1.89 )	1.038 ( 0.979–1.1 )	0.969 ( 0.905–1.038 )
	*p*	0.488	0.234	0.092	0.223	0.379
Heterogeneity	q-value	0.659691918	0.063639526	0.350562278	0.000820041	0.405272465
Pleiotropy	*p-*value	0.189750494	0.250334264	0.029533319	0.613853935	0.097439542

Inverse variance weighted (IVW), MR-Egger, weighted median, and weighted mode methods were used in this study. nSNPs, number single nucleotide polymorphisms; Total-BMD, total body bone mineral density; Heel-BMD, heel bone mineral density; CI, confidence interval

Then we examined various BMD measures including heel bone mineral density (Heel-BMD) and total bone mineral density (Total-BMD), utilizing them as exposures in a two-sample Mendelian randomization analysis for AS. Our analysis indicated a positive causal effect of Heel-BMD and AS (95% CI = 0.795 to 0.971, *p* = 0.012), and Total-BMD and AS (95% CI = 0.907 to 0.990, *p* = 0.017), as shown in Table 2.

**Table 2:** Causal effects of BMD (Heel-BMD, Total-BMD) on AS.

Exposures		Heel-BMD (nSNPs = 17)	Total-BMD (nSNPs = 8)
IVW	OR(95%CI)	0.879 ( 0.795–0.971 )	0.948 ( 0.907–0.990 )
	*p*	**0.012**	**0.017**
MR Egger	OR(95%CI)	0.898 ( 0.693–1.164 )	0.902 ( 0.82–0.992 )
	*p*	0.429	0.077
Weighted median	OR(95%CI)	0.813 ( 0.723–0.914 )	0.949 ( 0.896–1.005 )
	*p*	**< 0.01**	0.072
Weighted mode	OR(95%CI)	0.808 ( 0.685–0.953 )	0.953 ( 0.892–1.019 )
	*p*	**0.022**	0.200
Heterogeneity	*p*	0.051	0.484
Pleiotropy	*p*	0.860	0.292

Inverse variance weighted (IVW), MR-Egger, weighted median, and weighted mode methods were used in this study. nSNPs, number single nucleotide polymorphisms; Total-BMD, total body bone mineral density; Heel-BMD, heel bone mineral density; CI, confidence interval

**Fig. 2:**
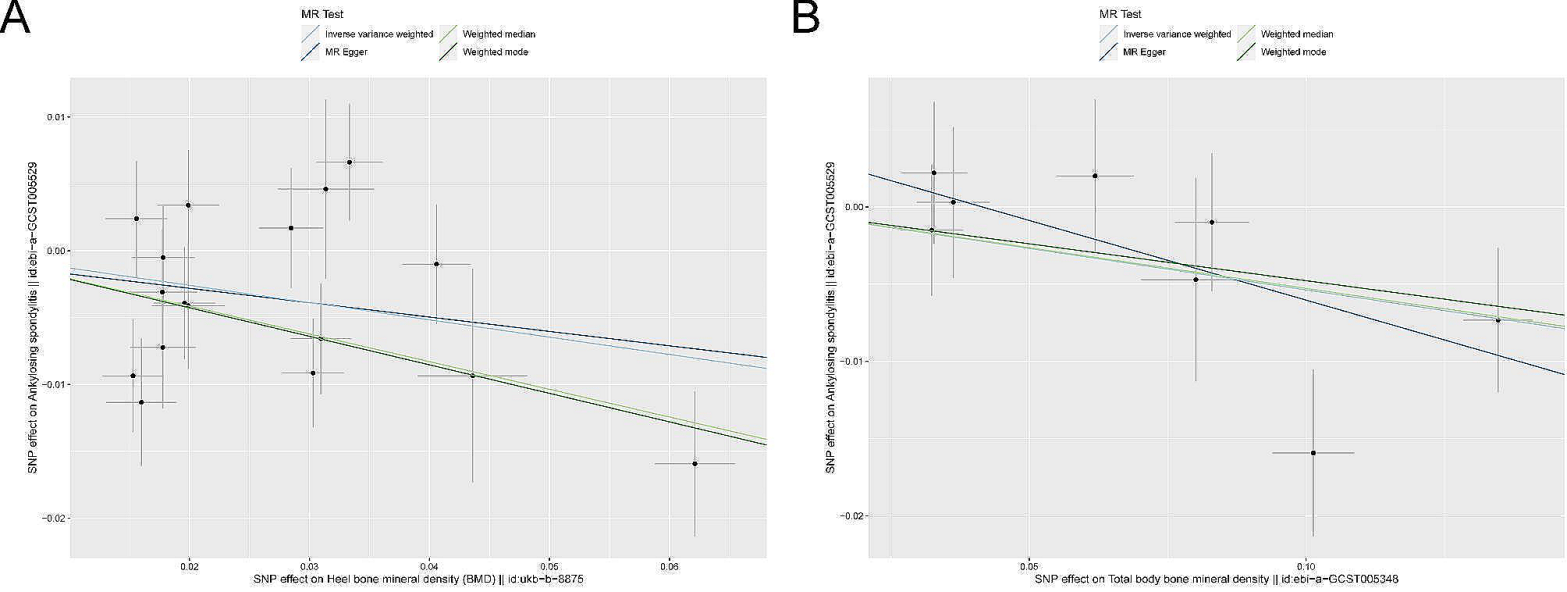
The scatter plot for MR analyses of causal associations between rheumatic diseases AS and BMD. (**A**) The scatter plot of the relationship between Heel-BMD and AS. (**B**) The scatter plot of the relationship between Total-BMD and AS.

To further clarify the correlation between Heel-BMD, Total-BMD, and AS, we conducted scatter plot analyses. Figure 2 demonstrate a negative correlation between Heel-BMD and AS (Fig. 2A), as well as Total-BMD and AS (Fig. 2B). These results suggest that an increase in Heel-BMD, and Total-BMD lead to a reduced risk of AS. Notably, no heterogeneity or pleiotropy was observed in these findings.

### Casual relationship between RA and BMD or fractures

**Fig. 3:**
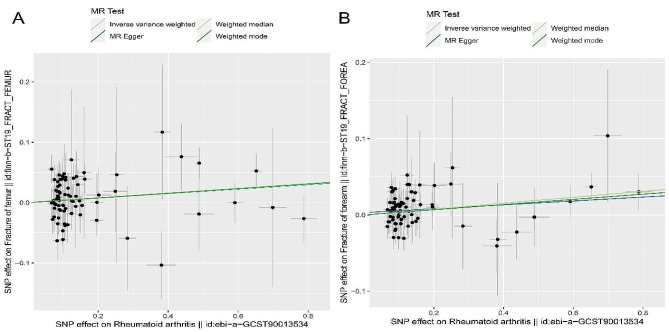
The scatter plot for MR analyses of causal associations between rheumatic diseases RA and fractures. (**A**) The scatter plot of the relationship between RA and fracture of femur. (**B**) The scatter plot of the relationship between RA and fracture of forearm

The MR results for RA with respect to BMD or fractures are displayed in Table 3. Cochran’s Q test showed the absence of heterogeneity and pleiotropy. Therefore, we adopted an IVW method to assess the causal relationship between RA and BMD or fracture risk [27, 28]. As shown in Table 3, the results showed a positive causal association between RA and fractures of the femur (95% CI = 1.0001 to 1.077, *p* = 0.046), as well as RA and fractures of the forearm (95% CI = 1.015 to 1.064, *p* < 0.01). These results suggest that RA can lead to an increased risk of both femur and forearm fractures without heterogeneity or pleiotropic effects. However, as shown in Table 4, there was absence of evidence for causal connection between fracture of forearm and RA (95% CI = 0.929–1.106, *p* = 0.759). To further elucidate the association between RA and fractures, we conducted a scatter plot analysis. As depicted in Fig. 3A and B, the slope of the scatter plot (β value) is greater than 0, suggesting that RA may elevate the risk of both femur and forearm fractures (Fig. 3).

**Table 3:** The Mendelian randomization (MR) analysis results of RA on fracture of forearm, fracture of femur, fracture of lumbar spine and pelvis, Heel-BMD and Total-BMD.

Outcomes		Fracture of forearm (nSNPs = 80)	Fracture of femur (nSNPs = 80)	Fracture of lumbar spine and pelvis (nSNPs = 80)	Heel-BMD (nSNPs = 80)	Total-BMD (nSNPs = 82)
IVW	OR(95%CI)	1.04 ( 1.015–1.064 )	1.038 ( 1.001–1.077 )	1.029 ( 0.985–1.075 )	0.992 ( 0.987–0.997 )	0.993 ( 0.98–1.007 )
	*p*	**< 0.01**	**0.046**	0.197	**< 0.01**	0.324
MR Egger	OR(95%CI)	1.026 ( 0.99–1.063 )	1.038 ( 0.981–1.098 )	1.021 ( 0.956–1.091 )	0.998 ( 0.99–1.006 )	1.006 ( 0.983–1.029 )
	*p*	0.162	0.200	0.533	0.578	0.615
Weighted median	OR(95%CI)	1.039 ( 1.003–1.077 )	1.041 ( 0.982–1.103 )	1.064 ( 1.002–1.13 )	0.998 ( 0.991–1.004 )	1 ( 0.981–1.02 )
	*p*	0.035	0.177	0.042	0.453	0.965
Weighted mode	OR(95%CI)	1.035 ( 1.005–1.065 )	1.039 ( 0.988–1.094 )	1.047 ( 0.993–1.103 )	0.996 ( 0.99–1.002 )	1.004 ( 0.987–1.02 )
	*p*	0.023	0.141	0.090	0.223	0.659
Heterogeneity	*p*	0.397	0.760	0.324	< 0.001	0.002
Pleiotropy	*p*	0.976	0.328	0.765	0.072	0.174

Inverse variance weighted (IVW), MR-Egger, weighted median, and weighted mode methods were used in this study. nSNPs, number single nucleotide polymorphisms; Total-BMD, total body bone mineral density; Heel-BMD, heel bone mineral density; CI, confidence interval

**Table 4:** Causal effects of fracture of forearm and BMD (Heel-BMD, Total-BMD) on RA.

Exposures		Fracture of forearm (nSNPs = 77)	Heel-BMD (nSNPs = 319)	Total-BMD (nSNPs = 77)
IVW	OR(95%CI)	1.014 ( 0.929–1.106 )	0.983 ( 0.931–1.039 )	0.975 ( 0.907–1.047 )
	*p*	0.759	0.549	0.486
MR Egger	OR(95%CI)	0.951 ( 0.703–1.286 )	0.972 ( 0.878–1.077 )	1.018 ( 0.835–1.241 )
	*p*	0.759	0.593	0.862
Weighted median	OR(95%CI)	1.006 ( 0.899–1.126 )	1 ( 0.905–1.105 )	1.022 ( 0.921–1.135 )
	*p*	0.915	0.999	0.681
Weighted mode	OR(95%CI)	0.997 ( 0.875–1.135 )	0.971 ( 0.883–1.067 )	1.155 ( 0.999–1.335 )
	*p*	0.964	0.541	0.056
Heterogeneity	*p*	0.442	0.279	0.149
Pleiotropy	*p*	0.682	0.800	0.649

Inverse variance weighted (IVW), MR-Egger, weighted median, and weighted mode methods were used in this study. nSNPs, number single nucleotide polymorphisms; Total-BMD, total body bone mineral density; Heel-BMD, heel bone mineral density; CI, confidence interval

### Casual relationship between SLE and BMD or fractures

**Fig. 4:**
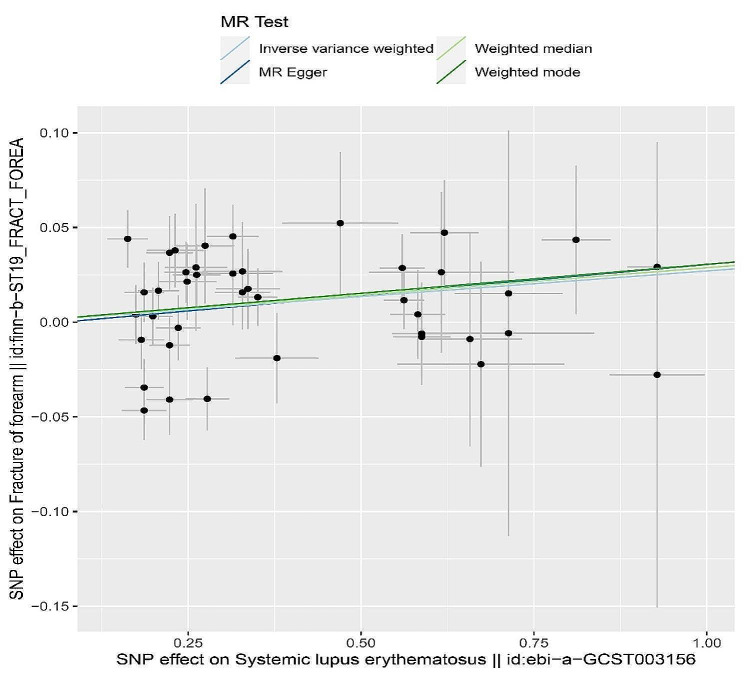
The scatter plot for MR analyses of causal associations between rheumatic diseases SLE and fracture of forearm

In addition, the correlation between SLE and BMD or fracture risk was also investigated as shown in Table 5. Based on our results, SLE exhibited a positive causal association with fracture of forearm (95% CI = 1.002 to 1.057, *p* = 0.034), suggesting that SLE can lead to an increased risk of fracture of forearm with significant heterogeneity and no pleiotropic effect (Table 5). Additionally, there was absence of evidence for causal connection between fracture of forearm and SLE (95% CI = 0.793–1.159, *p* = 0.516) (Table 6). Further scatter plot analysis demonstrates that SLE may also increase the risk of fracture of femur and fracture of forearm (Fig. 4).

**Table 5:** The Mendelian randomization (MR) analysis results used inverse variance weighted (IVW), MR-Egger, and weighted median methods showing causal effect of SLE on fracture of forearm, fracture of femur, fracture of lumbar spine and pelvis, Heel-BMD and Total-BMD.

Outcomes		Fracture of forearm (nSNPs = 41)	Fracture of femur (nSNPs = 41)	Fracture of lumbar spine and pelvis (nSNPs = 41)	Heel-BMD (nSNPs = 41)	Total-BMD (nSNPs = 42)
IVW	OR(95%CI)	1.035 ( 1.015–1.056 )	0.999 ( 0.969–1.029 )	1.032 ( 0.998–1.067 )	1.002 ( 0.998–1.006 )	1.006 ( 0.999–1.013 )
	*p*	< 0.01	0.931	0.067	0.312	0.080
MR Egger	OR(95%CI)	1.02 ( 0.977–1.065 )	1.022 ( 0.957–1.091 )	1.005 ( 0.933–1.081 )	0.995 ( 0.987–1.003 )	1.004 ( 0.99–1.019 )
	*p*	0.374	0.515	0.903	0.201	0.598
Weighted median	OR(95%CI)	1.032 ( 1.004–1.06 )	1 ( 0.96–1.042 )	1.042 ( 0.99–1.096 )	1 ( 0.996–1.005 )	1.007 ( 0.996–1.018 )
	*p*	0.024	0.991	0.114	0.897	0.199
Weighted mode	OR(95%CI)	1.031 ( 1–1.063 )	1.002 ( 0.948–1.059 )	1.058 ( 0.981–1.141 )	0.999 ( 0.994–1.004 )	1.006 ( 0.988–1.024 )
	*p*	0.060	0.938	0.152	0.761	0.522
Heterogeneity	*p*	0.153	0.158	0.563	0.003	0.581
Pleiotropy	*p*	0.436	0.457	0.424	0.044	0.748

Inverse variance weighted (IVW), MR-Egger, weighted median, and weighted mode methods were used in this study. nSNPs, number single nucleotide polymorphisms; Total-BMD, total body bone mineral density; Heel-BMD, heel bone mineral density; CI, confidence interval

**Table 6:** Causal effects of fracture of forearm and BMD (Heel-BMD, Total-BMD) on SLE.

Exposures		Fracture of forearm (nSNPs = 77)	Heel-BMD (nSNPs = 270)	Total-BMD (nSNPs = 67)
IVW	OR(95%CI)	1.122 ( 0.793–1.589 )	0.996 ( 0.859–1.154 )	1.055 ( 0.895–1.245 )
	*p*	0.516	0.958	0.522
MR Egger	OR(95%CI)	2.015 ( 0.705–5.761 )	1.043 ( 0.796–1.366 )	0.958 ( 0.623–1.473 )
	*p*	0.282	0.763	0.845
Weighted median	OR(95%CI)	1.183 ( 0.925–1.512 )	0.859 ( 0.688–1.071 )	0.923 ( 0.736–1.157 )
	*p*	0.180	0.177	0.487
Weighted mode	OR(95%CI)	1.181 ( 0.884–1.576 )	0.889 ( 0.716–1.103 )	0.887 ( 0.668–1.179 )
	*p*	0.323	0.286	0.412
Heterogeneity	*p*	0.051	< 0.001	0.032
Pleiotropy	*p*	0.333	0.693	0.634

Inverse variance weighted (IVW), MR-Egger, weighted median, and weighted mode methods were used in this study. nSNPs, number single nucleotide polymorphisms; Total-BMD, total body bone mineral density; Heel-BMD, heel bone mineral density; CI, confidence interval

## Discussion

This study employed extensive GWAS data from publicly available databases and conducted an unbiased two-sample MR analysis to investigate the causal associations between rheumatic diseases, BMD, and fracture risk. Following rigorous quality control measures, we obtained the following results: (1) There is no significant causal relationship between AS and either BMD or fracture risk; (2) RA may lead to an increased risk of fracture of femur and fracture of forearm, with no heterogeneity or multiplicity; (3) SLE may lead to an increased risk of fracture of forearm, with significant heterogeneity and no pleiotropic effects; (4) Higher Heel-BMD and Total-BMD are linked to a reduced risk of AS, without heterogeneity or pleiotropic effects; (5) There is no significant relationship between fractures and rheumatic diseases. This study found that RA and SLE can lead to an increased risk of fractures, while increased Heel-BMD and Total-BMD can lead to a reduced risk of AS. These findings provide new evidence for the treatment and prevention of clinical rheumatic diseases.

The relationship between rheumatic diseases and BMD is intricate and affected by multiple factors. AS, RA and SLE are autoimmune diseases, and osteoporosis has been recognized as a common additional manifestation in individuals with AS, RA, and SLE [21, 29–32]. Numerous studies have demonstrated that patients with AS [33–35], RA [36, 37], and SLE [19, 38] tend to have significantly lower BMD. However, whether there is a causal relationship between rheumatoid arthritis and BMD remains unclear. In the present study, we revealed that an increase in BMD including Heel-BMD and Total-BMD contributes to a diminished risk of AS without the presence of heterogeneity or pleiotropic effects. A recent study by Karberg et al., also reached conclusions consistent with our results. They encompassed 103 AS patients and found that bone loss was more prevalent in AS patients with syndesmotic osteophytes [39].

In addition, there are many studies on the impact of rheumatic diseases on fractures. A cohort study examining long-term fracture risk in rheumatoid arthritis over a 10-year period showed that RA-specific risk factors are important for future development of severe fragility fractures [8]. Another study based on current literature showed that childhood rheumatic disease is associated with reduced BMD and increased vertebral and nonvertebral fractures [40]. Here, we found that RA and SLE could elevate the risk of fracture of forearm. In a population-based cross-sectional study, the detection rate of self-reported vertebral fractures increased from 6.4 to 18.9% in women with RA and/or SLE, suggesting that RA and SLE contribute to fracture risk increase [41]. Our results, together with these previous findings, provide some theoretical basis for the clinical treatment of patients with RA or SLE, and doctors can implement necessary preventive measures to reduce the risk of fractures.

This study has the following innovations: (1) The investigation commences by delving into molecular mechanisms, employing rheumatic diseases as primary exposure factors, and scrutinizing the causal interplay between rheumatic diseases, BMD, and fracture risk. This approach is underpinned by robust theoretical foundations and holds significant clinical relevance. (2) Rigorous quality control measures and analytical techniques were applied in this study, employing a diverse set of models to assess causal effects. As a result, the research outcomes are both dependable and consistent. (3) In contrast to prior MR studies that typically focus on single exposure factors, this study encompasses three distinct, prototypical rheumatic diseases. This presented a considerable workload and analytical complexity, setting it apart in terms of scope and challenge from previous MR investigations.

This study has its own set of limitations: (1) The GWAS data for rheumatic diseases are derived exclusively from European populations. Consequently, the findings may not be extrapolated to other races and regions. More comprehensive studies are warranted among diverse ethnic groups; (2) Despite leveraging the largest available large-scale GWAS data, subsequent research endeavors should focus on further augmenting the sample size to yield more precise assessments. (3) The large number of SNPs used, while enhancing instrumental strength, does not fully preclude the risk of pleiotropy or violation of instrumental variable assumptions. (4) The multiple testing corrections applied might not completely eliminate the risk of Type I or II errors, particularly for small effect sizes. Therefore, findings should be interpreted with caution, keeping in mind the potential for both false positives and negatives, and the need for replication in more diverse populations to confirm these causal inferences.

## Conclusions

We utilized a two-sample MR approach to explore potential causal relationships between rheumatic diseases and BMD or fracture risk. Our analysis showed that RA and SLE are associated with an increased risk of fracture. Increased heel BMD and total BMD are associated with decreased risk of AS. In contrast, there was no significant relationship between fractures and rheumatic diseases. These insights shed light on the relationship between BMD and rheumatic diseases, highlighting that increased BMD may reduce the risk of developing AS.

### Electronic supplementary material

Below is the link to the electronic supplementary material.

Supplementary Material 1

Supplementary Material 2

## Data Availability

The data used to support the findings of this study are available from the corresponding author upon request.
