# Supplementary material for: Potential association of rheumatic diseases with bone mineral density and fractures: a bi-directional mendelian randomization study
Source: BMC Musculoskelet Disord. 2024 Jul 5;25:521. doi: 10.1186/s12891-024-07496-w (PMC11225327; doi:10.1186/s12891-024-07496-w)
Supplement: Supplementary file 1 — Supplementary Material 1 [file 12891_2024_7496_MOESM1_ESM.docx]

Supplementary table 1 Data sources used in this study.

| Trait | GWAS ID | Sample size | SNPs | Population |
| --- | --- | --- | --- | --- |
| Ankylosing spondylitis | ebi-a-GCST005529 | 22,647 | 99,962 | European |
| Rheumatoid arthritis | ebi-a-GCST90013534 | 58,284 | 13,108,512 | European |
| Systemic lupus erythematosus | ebi-a-GCST003156 | 14,267 | 7,071,163 | European |
| Heel bone mineral density | ukb-b-8875 | 265,627 | 9,851,867 | European |
| Total body bone mineral density | ebi-a-GCST005348 | 56,284 | 16,162,733 | European |
| Fracture of forearm | finn-b-ST19_FRACT_FOREA | 215,724 | 16,380,460 | European |
| Fracture of femur | finn-b-ST19_FRACT_FEMUR | 215,443 | 16,380,458 | European |
| Fracture of lumbar spine and pelvis | finn-b-ST19_FRACT_LUMBAR_SPINE_PELVIS | 215,698 | 16,380,457 | European |
